# Neovascular glaucoma: pathophysiology, diagnosis, and contemporary management

**DOI:** 10.3389/fmed.2026.1829049

**Published:** 2026-05-22

**Authors:** Danyang Yu, Chaoxiong Cui, Guanghao Li, Zhenbao Wang

**Affiliations:** 1 Qingdao Central Hospital, University of Health and Rehabilitation Sciences, Qingdao, Shandong, China; 2 Qingdao Eighth People's Hospital (East Campus), Qingdao, Shandong, China; 3 Qingdao Municipal Hospital, University of Health and Rehabilitation Sciences, Qingdao, Shandong, China

**Keywords:** anterior-segment imaging, anti-VEGF therapy, glaucoma drainage devices, neovascular glaucoma, panretinal photocoagulation, rubeosis iridis

## Abstract

**Background:**

Neovascular glaucoma (NVG) is a vision-threatening secondary glaucoma driven by pathologic neovascularization of the iris and anterior chamber angle and is most commonly secondary to ischemic retinal diseases. Despite advances in retinal imaging and anti-vascular endothelial growth factor (anti-VEGF) therapy, NVG remains difficult to manage and is associated with high rates of vision loss, ocular morbidity, and surgical failure.

**Purpose:**

This narrative review synthesizes the contemporary understanding of NVG pathophysiology, summarizes diagnostic approaches with emphasis on anterior-segment and retinal imaging, evaluates current medical and surgical management strategies, and highlights emerging directions for research and clinical innovation.

**Findings:**

NVG originates from retinal ischemia, which elevates intraocular VEGF and other proangiogenic and inflammatory mediators, inducing rubeosis iridis and fibrovascular membrane formation at the angle. Clinical staging ranges from early iris neovascularization with normal intraocular pressure (IOP) to advanced angle closure with refractory IOP elevation and painful blind eyes. Diagnosis requires careful slit-lamp examination and gonioscopy, supplemented by multimodal imaging: ultrawidefield fluorescein angiography quantifies retinal nonperfusion; and anterior-segment OCT and ultrasound biomicroscopy delineate membrane extent and angle architecture; anterior-segment OCTA is investigational for vascular mapping. Management relies on two parallel goals—rapid suppression of anterior segment neovascularization and definitive treatment of the underlying retinal ischemia. Intravitreal anti-VEGF injections rapidly regress neovascularization and improve surgical conditions, but are temporizing unless combined with panretinal photocoagulation (PRP) or other ischemia-directed therapies.

**Conclusions and future directions:**

Long-term success in NVG patients requires integrated, individualized care that couples antiangiogenic bridging, durable management of retinal ischemia, and optimized surgical strategies.

## 1 Introduction

Neovascular glaucoma (NVG) is a severe, vision-threatening form of secondary glaucoma characterized by pathologic neovascularization of the iris (rubeosis iridis) and fibrovascular membrane formation in the anterior chamber angle (1). It most commonly arises as a complication of ischemic retinal disorders—such as proliferative diabetic retinopathy (PDR) and ischemic retinal vein occlusion—but can also follow ocular ischemic syndrome, ocular tumors, or severe ocular trauma. Clinically, NVG ranges from early asymptomatic iris neovascularization with preserved intraocular pressure (IOP) to advanced, fibrotic angle closure with refractory IOP elevation, pain, and poor visual prognosis (2). Despite improvements in systemic disease management and ophthalmic care, NVG remains associated with substantial morbidity, complex multimodal treatment requirements, and high rates of surgical failure (3).

Recent advances—notably intravitreal anti-VEGF agents and enhanced retinal and anterior-segment imaging (ultra-widefield fluorescein angiography, anterior-segment OCT/OCT-A, and ultrasound biomicroscopy)—have improved the ability to detect, temporize, and plan interventions for NVG (4, 5). However, anti-VEGF therapy is largely temporary unless combined with durable treatment of the underlying retinal ischemia, and surgical outcomes continue to be compromised by inflammation and aggressive fibrosis (6). This narrative review aims to synthesize the current understanding of NVG pathophysiology, outline practical diagnostic and imaging strategies, summarize contemporary medical and surgical management options, and identify promising research directions and unmet clinical needs that could guide future investigations and improve patient outcomes.

## 2 Materials and methods

### 2.1 Literature search strategy

This narrative review was conducted to summarize contemporary knowledge on neovascular glaucoma, with emphasis on pathophysiology, diagnosis, and management. We searched PubMed, Embase, and Web of Science for English-language articles published primarily between January 2000 and March 2026, using combinations of the following terms: “neovascular glaucoma,” “rubeosis iridis,” “anti-VEGF,” “panretinal photocoagulation,” “glaucoma drainage device,” “trabeculectomy,” “cyclophotocoagulation,” “ultrawidefield fluorescein angiography,” “optical coherence tomography angiography,” and “ultrasound biomicroscopy.” We also manually screened the reference lists of relevant review articles and key original studies to identify additional articles of potential relevance.

Priority was given to randomized or comparative clinical studies, multicenter studies, high-quality observational studies, and recent review articles. Landmark older studies were included when needed for historical context or to clarify important mechanistic or clinical concepts. Articles were selected by the authors on the basis of title and abstract screening, followed by full-text review when appropriate. Because this is a narrative review, no formal quality scoring, risk-of-bias assessment, or meta-analysis was performed; instead, the literature was synthesized thematically, with attention to the strength, consistency, and clinical applicability of the available evidence.

### 2.2 Evidence synthesis and bias mitigation

To minimize biased evidence selection and reduce the risk of cherry-picking, the literature synthesis was intentionally broad. We aimed to capture both supportive and conflicting findings, including randomized trials, comparative studies, retrospective series, review articles, and selected landmark papers that shaped current understanding of neovascular glaucoma. When evidence was heterogeneous or controversial, we explicitly noted these inconsistencies in the text rather than preferentially emphasizing only one side of the literature. In addition, we compared findings across different study designs and clinical contexts to avoid overinterpreting isolated positive results. This approach was intended to provide a balanced narrative synthesis while still acknowledging the varying quality and limitations of the available evidence.

## 3 Epidemiology and common causes

NVG is an uncommon but serious complication of ocular ischemia. Its incidence varies by population and the prevalence of underlying ischemic retinal disease: reported rates are higher in regions with limited access to timely retinal care or poor systemic disease control (7). PDR and ischemic central retinal vein occlusion (CRVO) are the leading causes worldwide, together accounting for the majority of NVG cases (8, 9). Other notable etiologies include ocular ischemic syndrome due to carotid artery stenosis (10), severe ocular trauma (11), intraocular tumors that disrupt retinal circulation, and chronic, untreated peripheral retinal ischemia from advanced proliferative retinopathies. The risk of progression to NVG increases with the extent and duration of retinal nonperfusion, poor metabolic control (e.g., uncontrolled diabetes), delayed or incomplete panretinal photocoagulation (PRP), and coexisting vascular comorbidities.

Although NVG is relatively uncommon, it constitutes approximately 3–8% of secondary glaucomas seen at tertiary centers and has accounted for 1–2% of glaucoma procedures in large referral series, highlighting its disproportionate surgical burden (1). Among patients with proliferative diabetic retinopathy, annual NVG incidence ranges from 2.5 to 6% in well-monitored cohorts but may approach 10% or higher when metabolic control is poor or when PRP is delayed (12). Ischemic central retinal vein occlusion carries the highest absolute risk: up to 40–45% of untreated ischemic CRVO eyes develop anterior segment neovascularization and NVG within six months (9). In Ekiti State, Nigeria, NVG accounted for roughly 13% of glaucoma referrals, a burden attributed to delayed PRP and limited anti-VEGF access in that setting (13), while a tertiary U. S. center reported an increasing number of urgent NVG tube shunt surgeries, largely among poorly controlled diabetic patients (7). Quantitative imaging and systemic markers, such as the extent of peripheral nonperfusion on widefield OCTA and elevated stress hyperglycemia ratio, further stratify risk and may explain why certain eyes progress rapidly despite treatment (14).

Clinical and demographic patterns reflect these drivers: NVG is more frequently encountered in older adults with systemic vascular disease and in populations with a high prevalence of diabetes mellitus (15). Within diabetic cohorts, poor glycemic control, longstanding diabetes, and proliferative disease with extensive nonperfusion strongly predict NVG development. Similarly, eyes with ischemic CRVO—characterized by widespread capillary nonperfusion on fluorescein angiography—have substantially elevated NVG risk compared with nonischemic CRVO. Geographical and health-system factors (screening coverage, availability of PRP and anti-VEGF therapy) importantly modulate the realized incidence and outcomes, underscoring the role of preventive retinal care in NVG mitigation (13).

## 4 Pathophysiology

### 4.1 Angiogenic and inflammatory mediators

NVG reflects a complex interplay of angiogenic, inflammatory, and fibrotic processes rather than a single-pathway disease. VEGF is the dominant proangiogenic driver identified in aqueous and vitreous samples from affected eyes (16), but other growth factors and cytokines—placental growth factor (PlGF), fibroblast growth factors (FGFs), platelet-derived growth factor (PDGF), interleukins, and chemokines—contribute to the proangiogenic milieu (17). Hypoxic retinal cells (including Müller cells and retinal pigment epithelium), infiltrating inflammatory cells, and perhaps ischemic retinal microglia are important cellular sources of these mediators. The concentration gradients between the posterior segment and anterior chamber facilitate the diffusion of soluble factors through the vitreous and anterior hyaloid, accounting for the temporal relationship between retinal ischemia and anterior segment neovascularization. Quantitative correlations between aqueous VEGF levels, the extent of retinal nonperfusion on angiography, and the degree of iris/angle neovascularization have been observed in clinical studies, supporting a pathophysiologic continuum from retinal ischemia to anterior segment pathology (18).

However, the current mechanistic evidence should be interpreted with caution. Much of the available data is derived from small observational cohorts, cross-sectional aqueous humor analyses, or experimental models, which provide strong biologic plausibility but limited causal inference. Although VEGF appears to be the dominant driver of anterior-segment neovascularization (19), the relative contribution of inflammatory and profibrotic pathways likely varies across disease phenotypes and stages. This heterogeneity may partly explain why some eyes progress rapidly despite treatment, whereas others show slower or incomplete neovascular responses. Accordingly, NVG should be viewed as a biologically heterogeneous syndrome rather than a uniform VEGF-driven entity (1).

### 4.2 Fibrovascular membrane dynamics and fibrosis

The fibrovascular membranes that characterize NVG are cellular and dynamic structures rather than inert scar tissue. Endothelial cells form immature, leaky vessels that are supported by pericytes and an expanding network of activated stromal cells, including myofibroblasts derived from local fibroblasts, perivascular cells, or transdifferentiating endothelial/epithelial cells. These myofibroblasts express *α*-smooth muscle actin and generate contractile forces that, together with extracellular matrix deposition (collagens and fibronectin), produce progressive traction and synechial adhesion. Matrix metalloproteinases (MMPs) and their inhibitors regulate matrix turnover, whereas profibrotic cytokines such as TGF-*β* and connective tissue growth factor (CTGF) skew the tissue response toward fibrosis (20). This cellular plasticity and balance between proteolysis and matrix synthesis underlie the variable clinical behavior—rapid membrane contraction in some eyes versus more indolent progression in others (Table 1).

**Table 1:** Summary of clinical stage and stage-based management of neovascular glaucoma.

Clinical stage	Key clinical features	Main management priorities	When to consider surgery
Stage 1: Rubeosis iridis without elevated IOP	Fine iris or angle neovascularization; IOP normal or near normal	Prompt anti-VEGF as a bridge; urgent PRP; PPV with endolaser if media opacity prevents laser	Usually not required unless ischemia cannot be controlled or progression is rapid
Stage 2: Open-angle NVG	Iris/angle neovascularization with elevated IOP but limited PAS	Maximal tolerated medical therapy; anti-VEGF; prompt PRP or PPV/endolaser if needed	If IOP remains uncontrolled despite medical and ischemia-directed treatment
Stage 3: Synechial angle-closure NVG	Extensive PAS; chronic angle closure; refractory IOP elevation; pain	Ongoing ischemia control if possible; inflammation reduction; pain control	GDD is commonly favored; trabeculectomy in select eyes; cyclodestruction for poor visual potential or blind, painful eyes

IOP, intraocular pressure; anti-VEGF, anti-vascular endothelial growth factor; PRP, panretinal photocoagulation; PPV, pars plana vitrectomy; PAS, peripheral anterior synechiae; GDD, glaucoma drainage device; NVG, neovascular glaucoma.

### 4.3 Anatomic progression to synechial angle closure

Anatomically, neovascularization may begin at the pupillary margin or iris surface and extend to the angle, with early vessels often visible on slit-lamp examination as fine, frond-like vessels on the pupillary ruff or iris stroma (21). Gonioscopy can reveal new vessels crossing the trabecular meshwork and early membrane formation without synechiae; progressive membrane contraction leads to peripheral anterior synechiae (PAS), which may initially be focal but can coalesce to produce 360° synechial angle closure. Hemorrhage into the anterior chamber (hyphema) is frequent due to the fragility of neovessels and can further exacerbate inflammation and fibroproliferation, accelerating angle closure. These anatomic events translate into functional stages: (1) rubeosis with normal IOP, (2) open-angle NVG with early IOP elevation from trabecular dysfunction, and (3) chronic angle-closure NVG with extensive PAS and refractory IOP elevation.

### 4.4 Temporal dynamics and progression modifiers

The tempo of NVG development is variable and influenced by the magnitude of retinal ischemia, systemic vascular health, prior retinal treatments, and intercurrent events (22). Ischemic CRVO commonly results in rapid NVG onset within weeks to months, whereas the development of diabetic retinopathy may be more insidious but still accelerated by inadequate PRP or recurrent vitreous hemorrhage. Timely PRP, optimization of systemic conditions (glycemic, blood pressure, lipid control), and early intravitreal anti-VEGF can modify the course, reducing the incidence or delaying progression—but do not eliminate risk entirely. Clinically, earlier-stage NVG has better visual and surgical prognoses because the PAS burden is lower and the angle architecture remains more salvageable.

### 4.5 Therapeutic implications from pathobiology

The pathobiologic insights discussed above carry several practical therapeutic implications. Anti-VEGF agents are exceptionally useful as short-term ‘bridge’ therapy, but clinicians should recognize the phenotypes in which their effect is transient or insufficient (23). Eyes with established 360° peripheral anterior synechiae, pronounced fibrovascular contracture, recurrent hyphema, or unabated retinal nonperfusion (e.g., persistent large UWFA nonperfusion areas despite previous injections) often continue to show anterior-segment neovascular activity even after repeated anti-VEGF, underscoring that angiogenesis suppression alone cannot reverse the fibrotic remodeling (24). Similarly, patients with systemic markers of poor metabolic control, such as an elevated stress hyperglycemia ratio (SHR), which reflects acute hyperglycemia relative to baseline glycemic status, or those who cannot adhere to frequent injections are more likely to experience rebound neovascularization (12). For these subgroups, anti-VEGF should be explicitly framed as a temporizing measure while definitive ischemia-directed treatment and antifibrotic strategies are arranged. Quantitative anterior-segment imaging (OCTA vascular density, UBM membrane thickness) can help identify eyes with high membrane burden and guide the timing of angle surgery or antifibrotic intervention (25). Emerging antifibrotic approaches—such as intraoperative or sustained-release anti-TGF-*β*/CTGF agents, ROCK inhibitors, or combined antiangiogenic/antifibrotic implants—are particularly attractive for eyes with brisk membrane contraction or recurrent tube encapsulation (26–28). Long-acting anti-VEGF platforms or gene-based biologics may offset logistic barriers in high-risk populations and reduce the need for frequent retreatment (29). In short, avoiding treatment failure in NVG requires early recognition of angiogenic versus fibrotic dominance and the deliberate sequencing of antiangiogenic bridging, ischemia resolution, and fibrosis-targeting adjuncts.

## 5 Clinical presentation and examination

### 5.1 Spectrum of presentation

Patients with NVG present along a spectrum from asymptomatic iris neovascularization to painful, vision-threatening angle-closure glaucoma. Early-stage disease may be discovered on routine slit-lamp exams as fine, tortuous vessels on the pupillary margin or iris surface; symptoms are often minimal. As the IOP increases, patients may report ocular discomfort, photophobia, decreased vision, and red eyes (30). Hyphema from fragile neovessels can produce acute visual loss and markedly elevated IOP. On examination, conjunctival injection, corneal edema, mid-dilated nonreactive pupils (in advanced cases), and variable anterior chamber inflammation were observed. Careful gonioscopy is essential: early neovascular webs crossing the trabecular meshwork, ghost vessels, or focal PAS may be visible before diffuse angle closure. Tonometry frequently reveals elevated IOP, although IOP can be normal in the purely presynechial rubeosis stage. Posterior segment findings depend on the underlying cause—signs of PDR, extensive retinal hemorrhage or ischemia in CRVO, or evidence of ocular ischemic syndrome should be sought.

### 5.2 Diagnostic imaging and ancillary testing

Multimodal imaging is used to refine diagnosis, quantify ischemia, and guide therapy. Key modalities include the following:

- Slit-lamp photography and anterior segment photography were used to document the rubeosis and hyphema.
- Gonioscopy (dynamic and graded) remains the clinical gold standard for detecting angle neovascularization, membrane, and PAS.
- Anterior-segment OCT and ultrasound biomicroscopy (UBM) provide objective visualization of membrane thickness, angle architecture, iris root configuration, and ciliary body anatomy, which is useful when corneal edema limits gonioscopy.
- Anterior-segment OCTA is investigational but can noninvasively map the superficial iris and angle vasculature and may offer quantitative biomarkers in the future.
- Ultrawidefield fluorescein angiography (UWFA) accurately maps retinal nonperfusion and neovascular activity and helps determine the need for and extent of PRP.
- Optical coherence tomography (OCT) and OCTA of the posterior pole were used to assess macular ischemia, tractional changes, and retinal perfusion.
- B-scan ultrasonography is indicated when media opacity prevents fundus visualization to detect retinal detachment, vitreous hemorrhage, or intraocular masses.

Laboratory and systemic evaluations should be tailored to assess glycemic control (HbA1c), blood pressure, the lipid profile, carotid imaging when ocular ischemic syndrome is suspected, and vascular risk factor optimization. In atypical cases, hypercoagulability testing, inflammatory or infectious workup, and referral for systemic oncologic or vascular evaluation are considered.

Although multimodal imaging has improved diagnostic confidence and treatment planning, the evidence base for several modalities remains limited (4). Gonioscopy remains the clinical reference standard, but it is operator-dependent and may be difficult to perform in eyes with corneal edema, hyphema, or severe discomfort (31). UWFA is highly useful for quantifying retinal nonperfusion, yet standardized ischemic thresholds that reliably predict NVG onset or recurrence have not been universally established. Similarly, anterior-segment OCTA and other emerging vascular imaging techniques are promising, but they remain investigational because of limited external validation, variability in acquisition protocols, and uncertain correlation with long-term clinical outcomes. Thus, imaging should be interpreted as an adjunct to, rather than a substitute for, careful clinical assessment.

### 5.3 Staging and differential diagnosis

Several staging schemes exist that combine clinical, gonioscopic (32), and imaging findings; a pragmatic three-stage framework is useful for management decisions: (1) rubeosis iridis without IOP elevation, (2) open-angle NVG with iris/angle neovascularization and early IOP rise, and (3) chronic angle-closure NVG with extensive PAS and refractory IOP. Differential diagnoses include inflammatory or infectious iris neovascularization mimicking rubeosis, angle neovascularization secondary to intraocular tumors, neovascularization due to previous ocular surgery or chronic retinal detachment, and pseudoexfoliation or pigmentary glaucoma presenting with secondary angle changes. Distinguishing true ischemia-driven NVG from other causes can direct appropriate retinal, vascular, or oncologic workup and therapy.

Importantly, existing staging schemes are useful for communication and clinical decision-making, but they have not been fully standardized across studies. Different authors use varying combinations of slit-lamp findings, gonioscopic extent of neovascularization, PAS burden, and IOP level, which limits comparability between reports and complicates the interpretation of treatment outcomes. A more uniform, imaging-supported staging system may improve future prognostic studies and facilitate evidence-based treatment algorithms.

## 6 Management

### 6.1 Principles of management

**Figure 1:**
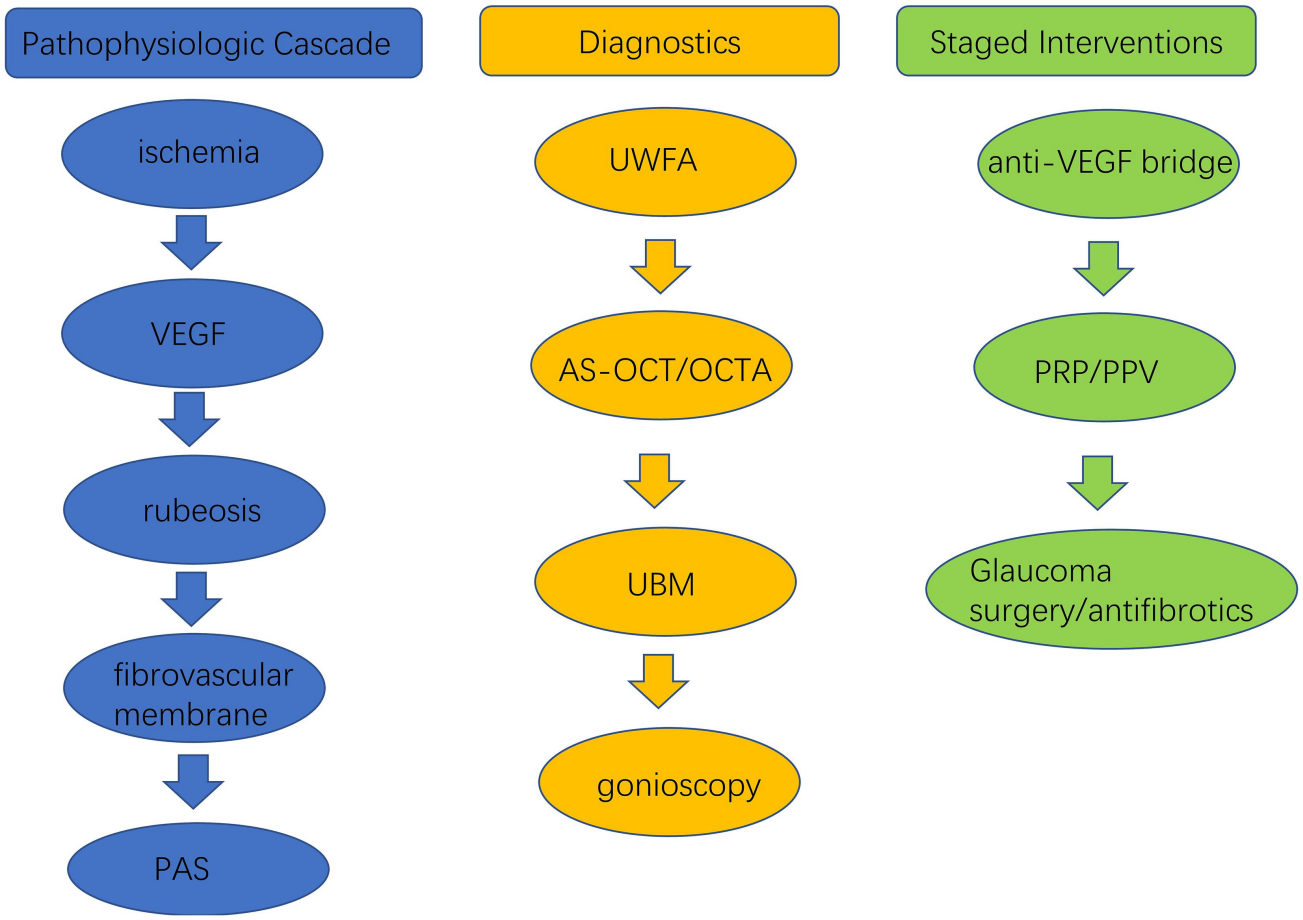
Pathophysiology-to-treatment cascade in neovascular glaucoma. VEGF, vascular endothelial growth factor; PAS, peripheral anterior synechiae; UWFA, ultra-widefield fluorescein angiography; AS-OCT, anterior segment optical coherence tomography; OCTA, optical coherence tomography angiography; UBM, ultrasound biomicroscopy; PRP, panretinal photocoagulation; PPV, pars plana vitrectomy.

Management of NVG requires a dual focus: rapid suppression of anterior-segment neovascular activity to reduce bleeding, inflammation, and membrane progression, and durable treatment of the ischemic stimulus together with reliable IOP control (33). In practice, urgent medical and intravitreal therapies serve as temporizing measures while definitive retinal ischemia treatment and, when needed, glaucoma surgery are undertaken. All decisions should be individualized according to disease stage, visual potential, comorbidities, and logistical realities, and systemic vascular risk factors should be optimized in parallel. Figure 1 frames the staged management pathway described below, helping orient clinicians to the evolving diagnostic and therapeutic priorities in NVG.

### 6.2 Acute medical stabilization

Acute medical management focuses on rapid optic nerve protection while the posterior and anterior segment teams prepare definitive therapy. Topical IOP-lowering agents (beta-blockers, carbonic anhydrase inhibitors, alpha-agonists, and prostaglandin analogs when appropriate), oral acetazolamide, and, in severe cases, hyperosmotics such as IV mannitol are used to reduce pressure rapidly (31). Intensive topical corticosteroids and cycloplegia reduce anterior chamber inflammation and pain and may limit early synechiae formation; anterior chamber paracentesis can be a temporizing measure when very high IOP threatens optic nerve perfusion or when corneal edema must be relieved to permit PRP. Management of hyphema follows standard principles (head elevation, cycloplegia, topical steroids, IOP control), with surgical washout reserved for persistent or vision-threatening hemorrhage. Escalate to paracentesis when IOP remains >40 mmHg despite maximal topical and systemic therapy or when corneal edema prevents laser delivery.

### 6.3 Intravitreal anti-VEGF agents as bridge therapy

Anti-VEGF injections serve primarily as short-term bridge therapy, particularly when anterior segment neovascularization is active, but definitive retinal treatment is pending (34–40). Rapid regression of neovessels reduces intraoperative bleeding and improves surgical visibility, yet clinicians should recognize that eyes with extensive PAS, pronounced iris/angle fibrosis, recurrent hyphema, or persistent retinal nonperfusion often redevelop neovascularization unless the underlying ischemia is treated. The VEGF trial reinforces this by showing that aflibercept-induced regression is transient without concurrent ischemia-directed therapy (29). Deliver anti-VEGF close to planned PRP/PPV or surgery (typically within 3–7 days) to maximize hemostatic benefit while not delaying definitive treatment, and avoid repeating injections without simultaneously addressing retinal ischemia.

### 6.4 Definitive retinal ischemia treatment

Definitive control of retinal ischemia is the cornerstone that converts the anti-VEGF bridge into durable disease suppression. Prompt, often intensive PRP should be delivered when media clarity allows (41, 42). When vitreous hemorrhage or opaque media prevent effective laser, early PPV with endolaser clears the visual axis and allows complete panretinal treatment in the same sitting. Coordination between retina and glaucoma teams is essential to optimize sequencing so that PRP/PPV precedes or coincides with glaucoma surgery whenever possible, minimizing the need for repeat interventions. If PRP is incomplete after two sessions or if nonperfusion extends into the far periphery on UWFA, prioritize PPV with endolaser and consider supplemental targeted laser postoperatively.

### 6.5 Surgical options for IOP control

Surgery becomes necessary when medical and retinal therapies cannot achieve an acceptable IOP. Glaucoma drainage devices (GDDs) are commonly favored in NVG because they bypass compromised trabecular outflow and often yield more reliable mid-term pressure control than trabeculectomy in eyes with aggressive conjunctival scarring and fibrosis (43). Trabeculectomy with mitomycin-C may remain appropriate in eyes with limited PAS (<180°) and healthy conjunctiva but carries a higher failure risk in NVG (35). Cyclodestructive procedures are reserved primarily for blind painful eyes or those that have failed multiple surgeries, balancing IOP lowering with the risk of hypotony and phthisis (44). Choose GDD when PAS is extensive or fibrosis is anticipated; reserve trabeculectomy for early-stage eyes with open angles and minimal conjunctival scarring, and document visual potential before selecting cyclodestruction. The available comparative evidence is limited and largely retrospective, so procedure selection should be individualized based on ocular surface status, PAS extent, and prior interventions.

### 6.6 Adjunctive antifibrotic strategies

Fibrosis and bleb encapsulation often dictate long-term failure, so adjunctive antifibrotic strategies should be considered when perioperative risk is high. Research is exploring sustained-release anti-VEGF implants, combined antiangiogenic/antifibrotic delivery, anti-TGF-*β* or anti-CTGF agents, ROCK inhibitors, and other modulators of myofibroblast activity and matrix remodeling (28, 45, 46). Improved anterior-segment imaging biomarkers (OCT/OCT-A metrics of membrane burden and vascularity) can help quantify fibrotic risk and time to interventions. Apply antifibrotic adjuncts (e.g., intraoperative mitomycin-C, sustained-release immunomodulators, or ROCK inhibitors) for eyes with high membrane thickness on UBM or for tubes showing early encapsulation, and use OCTA-derived vascular density to monitor response.

### 6.7 Stage-based practical approach

A stage-based approach translates the above interventions into a practical decision tree.

- Stage I (rubeosis with normal IOP): Use anti-VEGF as a bridge, initiate PRP, intensify systemic risk factor control, and monitor closely for progression.
- Stage II (open-angle NVG with IOP elevation): Combine anti-VEGF with expedited PRP/PPV, consider early surgical consultation if IOP remains >25 mmHg despite maximal medical/retinal therapy, and reassess PAS progression with gonioscopy or UBM.
- Stage III (chronic angle-closure with extensive PAS): Prioritize GDD (or cyclodestruction in eyes with poor visual potential), employ antifibrotic adjuncts based on preoperative fibrosis markers, and maintain aggressive IOP control with combination therapy.

Follow-up must be frequent and long-term, with readiness to repeat anti-VEGF or PRP for recurrent neovascularization and ongoing retinal surveillance. Because randomized head-to-head trials comparing staged versus combined interventions are lacking, local expertise, media clarity, and systemic comorbidities will continue to shape the exact sequence of anti-VEGF, PRP/PPV, and surgery.

### 6.8 Multidisciplinary care and patient counseling

Finally, optimal NVG care demands multidisciplinary coordination between glaucoma and retina specialists and active management of systemic vascular disease (47). Patient counseling should emphasize the need for multiple interventions, guarded visual prognosis when presentation is advanced, and the critical role of systemic risk-factor control to reduce recurrence and preserve remaining vision.

The outcomes and prognoses of NVG patients remain guarded and highly variable, reflecting heterogeneity in etiology, stage at presentation, and timeliness of definitive retinal and glaucoma care (48). The visual prognosis is principally determined by the status of the posterior segment at presentation: eyes with preserved macular perfusion and prompt PRP/PPV have a substantially better chance of retaining useful vision than eyes that present with extensive macular ischemia, dense vitreous hemorrhage or long-standing angle closure (49). IOP control after surgery is achievable in many eyes, particularly those with GDDs, but long-term success rates are lower than those for primary glaucomas because of aggressive subconjunctival and intraocular fibrosis leading to encapsulation and progressive failure. Reported series show meaningful proportions of eyes requiring multiple procedures, repeat anti-VEGF or adjunctive interventions; nonetheless, appropriately timed multimodal therapy can salvage vision and alleviate pain in a substantial subset of patients.

Complications are common and can threaten both vision and the integrity of glaucoma surgery (50–52). Hyphema from fragile neovessels can recur and exacerbate inflammation, corneal blood staining, and IOP spikes; intraoperative bleeding complicates dissection and increases the likelihood of postoperative scarring. Tubes and shunts carry risks of hypotony, tube exposure, corneal endothelial decompensation, and infection; exposure, in particular, predisposes patients to endophthalmitis and often necessitates revision with conjunctival repair or tube repositioning. Trabeculectomy in NVG patients is especially susceptible to early failure from conjunctival fibrosis and intraoperative hemorrhage. Cyclodestructive procedures can control IOP and pain in refractory eyes but may precipitate prolonged uveitis, chronic hypotony, and even phthisis bulbi in some cases. These risks emphasize the need for perioperative anti-VEGF therapy to reduce vascular friability, meticulous surgical techniques, aggressive antifibrotic strategies when appropriate, and close postoperative surveillance.

Several controversies persist in NVG management because high-quality comparative evidence is limited (53, 54). The optimal timing and frequency of anti-VEGF injections—how many doses are needed pre-operatively and whether scheduled repeats improve outcomes versus as-needed administration—remains debated. The choice between valved and nonvalved drainage devices lacks definitive superiority data in NVG specifically, and surgeon experience and patient anatomy often drive device selection. Whether combined procedures (simultaneous PPV/PRP with tube implantation) confer better overall outcomes than staged approaches is unclear: combined surgery reduces the number of anesthetic episodes and may accelerate definitive treatment, but can increase intraoperative complexity and postoperative inflammation. The threshold for employing cyclodestruction earlier versus reserving it for eyes with poor visual potential is also center-dependent. These gaps highlight an unmet need for randomized trials comparing staged versus combined strategies, device types, and anti-VEGF protocols, specifically in ischemia-driven NVG.

Research priorities that could meaningfully change practice include therapies that target the fibrotic arm of NVG pathobiology. Agents that antagonize TGF-*β*, CTGF, or other profibrotic mediators, delivered intraoperatively or via sustained-release systems, might reduce membrane contraction and bleb encapsulation and thereby extend the longevity of conventional surgeries and drainage implants (26, 27, 55). Long-acting intraocular anti-VEGF implants or gene-therapy approaches to provide more durable suppression of angiogenic signaling are attractive for patients with recurrent neovascularization who face logistic barriers to frequent injections. Translational studies of ROCK inhibitors, anti-matrix metalloproteinase strategies, and cell-based modulation of wound healing are underway in other fibrotic ocular contexts and may apply to NVG. Parallel advances in imaging—quantitative anterior-segment OCT/OCT-A metrics, standardized UWFA ischemia indices, and objective membrane characterization—would enable earlier detection, better staging, and more rigorous outcome measures for trials.

Practical measures in contemporary practice should emphasize rapid interdisciplinary coordination, early use of anti-VEGF as a temporizing measure, and prompt definitive retinal ischemia treatment whenever possible. Preoperative optimization—timing anti-VEGF injections within a window that reduces bleeding without compromising retinal therapy, using intraoperative antifibrotics judiciously, and planning techniques to minimize conjunctival trauma—can improve surgical conditions and may modestly improve outcomes (56). Clear patient counseling about the likelihood of multiple interventions, the guarded visual prognosis in advanced disease, and the importance of systemic vascular control is essential for informed decision-making and adherence to follow-up (57, 58).

In conclusion, NVG is a complex, multifactorial condition in which rapid angiogenic activity and progressive fibrosis threaten vision. Modern management hinges on a staged, multidisciplinary approach that combines urgent anti-VEGF therapy and medical therapy, definitive retinal ischemia control, and appropriately selected glaucoma surgery, with GDDs commonly favored for refractory cases. Despite advances, long-term outcomes are limited by fibrotic failure mechanisms and by the extent of posterior-segment damage at presentation; therefore, research into antifibrotic strategies, sustained therapeutic delivery, and improved imaging biomarkers represents the most promising path to improve patient outcomes.

## 7 Future directions

Looking forward, meaningful progress in NVG will require more sensitive standardized tools for early detection and risk stratification, together with translational studies of sustained-release therapies, antifibrotic strategies, and validated imaging biomarkers. Pragmatic randomized trials and prospective registries are needed to compare treatment sequencing, evaluate long-term outcomes, and improve real-world effectiveness and equity of care. Priority translational goals include the development and clinical testing of sustained-release anterior-segment anti-VEGF platforms and combined antiangiogenic/antifibrotic delivery systems (e.g., targeted anti-TGF-*β*/CTGF agents, ROCK inhibitors) to suppress both neovascular drive and membrane contraction; parallel evaluation of gene-based or longer-acting biologics could reduce the treatment burden for high-risk patients. Validation of quantitative anterior-segment imaging biomarkers (OCT/OCT-A metrics, membrane thickness/contractility indices) and standardized UWFA ischemia scores will be critical for refining staging, guiding the timing of intervention, and enabling objective endpoints in trials. Pragmatic randomized trials are urgently needed to compare the timing and sequencing of anti-VEGF, PRP/PPV, and glaucoma surgery (including device type and combined versus staged approaches), and to evaluate antifibrotic adjuncts to improve long-term device/bleb survival. Complementary advances in AI-enabled screening and telemedicine could promote earlier detection, especially in underserved settings, whereas prospective registries and patient-centered outcome research should assess real-world effectiveness, safety, and cost-effectiveness across diverse health systems (59, 60). Finally, multidisciplinary care pathways that integrate retina, glaucoma, and systemic vascular management—coupled with clear patient education and shared decision-making—will be essential to translate scientific advances into better, equitable outcomes for patients with this devastating condition.

A major limitation of the current literature is that much of the evidence comes from retrospective, single-center studies with short follow-up, variable inclusion criteria, and inconsistent outcome definitions (39). This makes it difficult to compare findings across studies or to determine the true incremental benefit of one treatment strategy over another. Future trials should therefore prioritize standardized staging criteria, uniform imaging endpoints, and clinically meaningful long-term outcomes such as sustained IOP control, repeat intervention rates, visual function, and quality of life.

## 8 Conclusion

Overall, the current NVG literature supports a biologically coherent treatment strategy, but the strength of evidence remains uneven across diagnostic and therapeutic domains, with many key decisions still based on lower-level observational data and expert consensus.

NVG is a complex, vision-threatening condition driven by ischemia-induced anterior-segment angiogenesis and subsequent fibrotic membrane formation. Because both angiogenesis and fibrosis contribute to disease progression, durable control rarely results from a single intervention. Effective management, therefore, requires a dual, stage-adapted strategy that both suppresses active neovascularization quickly and addresses the underlying retinal ischemic drive while restoring or bypassing impaired aqueous outflow.

In clinical practice, this approach involves the use of intravitreal anti-VEGF and aggressive IOP-lowering measures as rapid bridging therapies to reduce vessel friability, inflammation, and intraoperative bleeding, followed as soon as possible by definitive retinal treatment (PRP or PPV with endolaser when media are opaque). When medical and retinal measures cannot control IOP, timely glaucoma surgery—most commonly a drainage device in the NVG setting—should be performed with perioperative steps to minimize bleeding and scarring (preoperative anti-VEGF, careful conjunctival handling, and judicious use of antifibrotics). Close, frequent follow-up with readiness to repeat anti-VEGF or additional laser therapy is essential.

The prognosis is highly variable and is determined largely by the extent of posterior segment damage and the degree of synechial angle closure at presentation. Long-term failure is commonly driven by aggressive fibrosis, making antifibrotic strategies and sustained-release antiangiogenic approaches important priorities for research. Clear patient counseling about the likelihood of multiple procedures, guarded visual outcomes in advanced disease, and the need for systemic vascular risk-factor control is critical to optimize adherence and outcomes.
